# Measuring central coherence and set shifting in anorexia nervosa: the Navon Figures Task

**DOI:** 10.1186/2050-2974-2-S1-O45

**Published:** 2014-11-24

**Authors:** Jenna Blumberg, Karina Allen, Sue Byrne

**Affiliations:** 1 The University of Western Australia, Perth, Australia

## 

People with eating disorders (EDs) are reported to have set-shifting difficulties, strengths in local or detailed information processing and weak central coherence or global processing. This study aimed to explore the validity of a novel, brief, global, neurocognitive task which has not previously been utilised in an ED population, the Navon Figures Task (NFT). The NFT is substantially quicker to administer than traditional neurocognitive tasks. Twenty women with anorexia nervosa (AN) and 20 healthy control women completed the Rey-Osterrieth Complex Figures Task (RCFT) to measure global/local processing strategies and completed the Wisconsin Card Sorting Test (WCT) to measure of set-shifting ability. The NFT was used as a measure of both set-shifting ability and central coherence. Consistent with previous findings, women with AN showed a tendency towards poorer performance on the RCFT and WCT than healthy control women. Scores on the NFT and RCFT were significantly, positively and strongly correlated in both the AN and healthy control samples, as were scores on the NFT and WCT. This indicates that the NFT has good concurrent validity with the other measures. These preliminary findings support the use of the NFT as a sensitive and practical measure in assessing central coherence and set-shifting abilities in EDs. Using measures like the NFT may potentially enhance our understanding of neurocognitive traits in AN and may help guide our treatment approaches to AN in the future.

This abstract was presented in the **Assessment** stream of the 2014 ANZAED Conference.

